# A Clinical-Anatomical-Radiological Study of Extraperitoneal Spaces: A Case Series

**DOI:** 10.7759/cureus.56149

**Published:** 2024-03-14

**Authors:** Giridhar Ashwath, Eshwar Kathiresan Manasijan, Logeshbalaji Seelampatti Palanisamy, Anthony P Rozario, Nachiket Shankar

**Affiliations:** 1 General Surgery, St John's Medical College Hospital, Bengaluru, IND; 2 Anatomy, St John's Medical College Hospital, Bengaluru, IND

**Keywords:** umbilicovesical fascia, pre-vesicle space, peri vesicle space, presacral space, pararectal space, pelvic extraperitoneal space

## Abstract

Complications can arise secondary to anorectal suppurative diseases, with infections spreading along the extraperitoneal space, such as the peri-vesical, prevesical, pre-sacral, and pararectal spaces, resulting in abscesses at remote sites, which can make diagnosis more challenging. Due to the absence of peritonitis symptoms, there is a delay in presentation among such patients. Comprehending the intricacies of these areas and the way infection can spread within them is crucial for promptly identifying and effectively draining the extraperitoneal abscess.

We present a case series of six patients with a mean age of 45, all males. A total of three patients had undergone incision and drainage after being diagnosed with anorectal suppurative disease and remained symptomatic after the initial surgical intervention of incision and drainage. Two patients initially diagnosed with anterior abdominal abscesses patients, after being treated with incision and drainage, continued to have purulent discharge from the drainage site. Finally, the last patient continued to present with perianal pain after an open hemorrhoidectomy. CT scans of all six patients showed collections in the extraperitoneal spaces correlated with the observed complications.

To deepen our understanding of pelvic extraperitoneal spaces, cadaver dissections were conducted and compared with CT images. Through cadaver dissections and CT imaging, the study provides insights into the anatomy and interconnections of pelvic extraperitoneal spaces, emphasizing the importance of early CT scans for diagnosis. Understanding these intricate anatomical structures is essential for accurate diagnosis and efficient and effective treatment. Timely diagnosis is vital to prevent prolonged illness and reduce the risk of complications and mortality. The importance of early CT scans in suspected patients is underscored, which is highly important to expedite appropriate actions.

## Introduction

The extraperitoneal abscess arising in the pelvis can spread along its communication and present as an abscess in a distant location, making it difficult for the clinician to identify the source of infection. This extraperitoneal abscess causes insidious, occult, and prolonged illness. Diagnostic delay and inadequate drainage are common, resulting in prolonged sepsis and associated high morbidity and mortality [1]. An extraperitoneal abscess may not present with features of peritonism [2], and also, these patients are paucisymptomatic due to the deep location of the infection and the fact that the inflammatory response of the retroperitoneum is more limited than that of the peritoneum to infection [3]. In these settings, that is, in patients with ongoing sepsis, there should be a high index of suspicion of the extraperitoneal spread of infection, and imaging modalities like CT of the abdomen and pelvis should be obtained for early recognition to determine the extent of spread of extraperitoneal abscess. Extraperitoneal surgical drainage is the best treatment modality for these patients. So, understanding the anatomy of pelvic extraperitoneal spaces and their communication is important in clinical practice for efficient patient management. We report patients who have presented with extraperitoneal sepsis and made an attempt to understand the anatomy of pelvic extraperitoneal spaces using cadaveric dissection and correlating with CT imaging.

## Materials and methods

Approval for the study was obtained from the Institutional Ethics Committee (IEC) bearing reference number 65/2023 at St. John's Medical College Hospital, following which informed consent was obtained from patients involved in the study. Owing to the retrospective design, the data analysis began with the review of the data that had been digitally recorded from May 2022 and continued through June 2023. Furthermore, the electronically stored clinical data containing the patients' demographics, medical history, comorbidities, clinical symptoms, and history of prior surgical interventions were assessed. The procedure of the data extraction was supervised by a number of reviewers to guarantee accuracy. Additionally, cadaveric specimens were employed for the purposes of anatomical correlation and performing procedural practice. These specimens were acquired from the College's repository. While utilizing these specimens, the Department of Anatomy had oversight for the acquisition and preservation of the specimens, and they also ensured that established procedures were followed. When dissections were performed, it was ensured that as much of the specimen was preserved as possible. The research team reviewed the medical records of all patients diagnosed with ischiorectal/ perianal abscesses. This includes six patients who were all males between the ages of 35 and 50, and their details are presented in the subsequent section. The patients diagnosed with extraperitoneal abscesses on CT imaging and clinically diagnosed with ischiorectal or perianal abscesses were subsequently included in the cohort. The extraperitoneal abscess is defined in our study by evidence of collection or air foci in the pelvic extraperitoneal spaces such as the prevesicle, the para rectal, and the pre-sacral spaces, and there may be extensions of the collection into the anterior extraperitoneal space or the retroperitoneum.

## Results

Among the six patients who presented with extraperitoneal sepsis, for our understanding concerning the symptomatology and CT images, we would like to categorize them into A, B, and C. List A comprises three patients who had perianal pain as their presenting complaint and had undergone incision and drainage but remained symptomatic with persisting discharge in whom the CT images showed collection in prevesical and pararectal spaces ascending into pre peritoneum and retroperitoneum. Likewise, list B covers a patient who had recently undergone an open hemorrhoidectomy and presented with pain in the perianal region. The CT images showed air foci in the pararectal space extending into the retroperitoneum and mediastinum. Finally, list C included two patients who presented with features suggestive of anterior abdominal abscess and underwent incision and drainage, but they continued to have purulent discharge from the drainage site while CT imaging in them showed a collection in the pre-peritoneum extending into prevesical space. 

**Figure 1:**
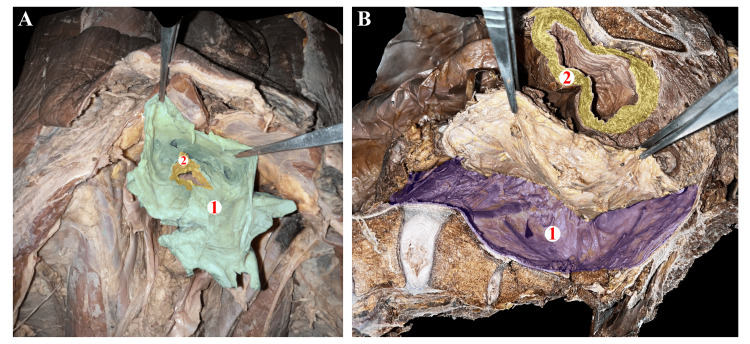
Images of the dissection of a male cadaver depicting structures in the pelvic region Figure 1A – Cadaver-oriented craniocaudal over the dissection table. The forceps are being used to hold the (1) Umbilicovesical fascia surrounding the (2) urinary bladder. Figure 1B – Dissection in the sagittal plane of the cadaver-oriented caudocranial over the dissection table, with the right and left sides of the image denoting the cranial and caudal ends, respectively. The dissection exposes (1) The presacral space posterior to which is the sacrum, (2) The urinary bladder and the forceps are being used to indicate the rectum.

**Figure 2:**
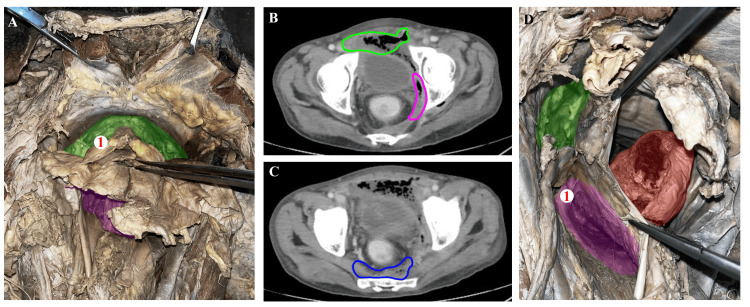
Correlating findings from the male cadaver with CT findings of patients Figure 2A – Cadaver-oriented craniocaudal over the dissection table exposing the (1) The prevesical space, highlighted in green, that lies anterolateral to the urinary bladder (pointed using forceps), Figure 2B – Axial contrast-enhanced CT of the abdomen showing a collection in the pararectal space (encircled in pink) extending cranially along the left lateral pelvic wall, involving the pre vesical region (encircled in green) with mild thickening of the urinary bladder wall, Figure 2C – Axial contrast-enhanced CT of the abdomen showing a collection in the presacral space (encircled in blue) Figure 2D – Cadaver-oriented craniocaudal over the dissection table and the specimen is being visualized in the left superolateral view, where prevesical space is highlighted in green, rectum is highlighted in red, (1) left pararectal space which lies lateral to the rectum and below the peritoneum is highlighted in purple.

Through cadaver dissections, the anatomy of the extraperitoneal spaces, such as the pre-sacral space (Figure 1B-1), prevesical space (Figure 2A-1), and the para rectal space (Figure 2D-1), was identified. The anatomy and the relationship of the umbilicovescial fascia (Figure 1A-1) with the urinary bladder (Figure 1A-2) is determined and is similar to the renal fascia, which envelops the kidney to form the perirenal space. The area in front of the umbilicovescial fascia is the prevesical space (Figure 2A-1) and lies anterolateral to the urinary bladder. In the course of cadaver dissection, we discovered the presence of loose areolar tissue, often called 'angel hairs' among colorectal surgeons, confirming the appropriate plane of dissection. Additionally, it is well-known that infections tend to spread along the path of least resistance, and it is possible that the presence of this loose areolar tissue between the spaces may contribute to the transmission of infections. Although the dissection did not provide evidence of intercommunication between the spaces, the CT images demonstrate the proof of communication through the travel of infections, and this validates what anatomy suggests, pathology proves.

Patient 1

**Figure 3:**
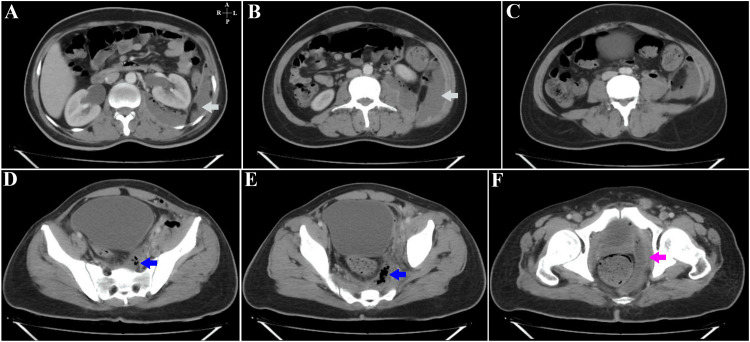
Patient 1- Contrast-enhanced CT images of the abdomen in the axial plane Figure 3A – At the level of the L3-L4 intervertebral disc space, showing a peripherally enhancing collection (cyan arrow) in the posterior, left lateral extra-peritoneal space, extending posterior to the descending colon and the left kidney. Figure 3B – Through the body of the L4 vertebra, an extension of the same collection (cyan arrow) is seen with fascial thickening and abutment of the left transversus abdominis. Figure 3C – At the inferior margin of the L4 vertebral body. Air foci are seen within the collection with subtle stranding of surrounding fat. Figure 3D – At the level of the S2-S3 vertebrae. Air foci (blue arrow) are seen within the collection with stranding of the retro-vesical fat and abutment of the left psoas major muscle. Figure 3E – At the level of the 4th and 5th sacral vertebrae. The air-containing collection (blue arrow) is seen in the left posterior extra-peritoneal rectal space. Thickening of the mesorectal fascia is seen with regional fat stranding. Figure 3F – A peripherally enhancing air-containing collection (pink arrow) is noted in the left hemi-pelvis at the level of the pubic symphysis. The obturator internus is seen laterally, and the rectum is present medial to the collection.

A 38-year-old male with type 2 diabetes mellitus was diagnosed with an ischiorectal abscess and initially underwent incision and drainage but presented to the emergency department with pain on the left side of his abdomen in septic shock two weeks later. On examination, the abdomen appeared soft and had no evidence of peritonitis, and a digital rectal examination revealed the presence of purulent discharge and tenderness at the operative site (Figure 3).

Patient 2

**Figure 4:**
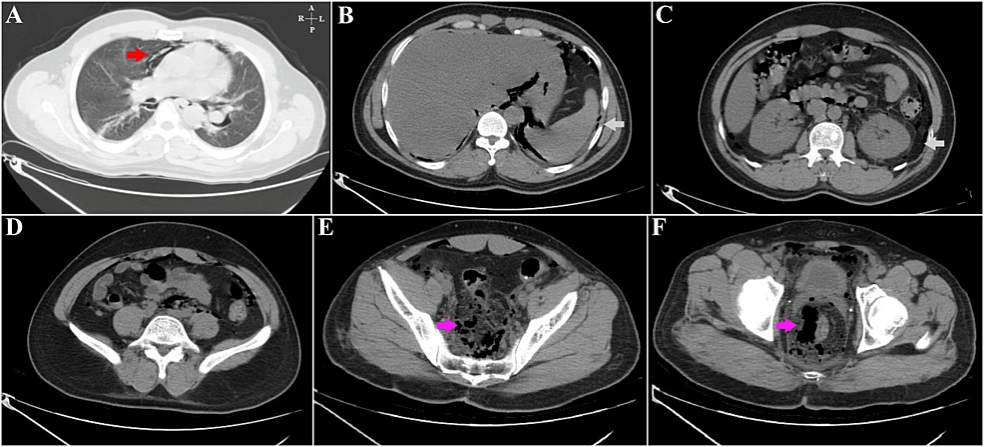
Patient 2 - CT images of the abdomen in the axial plane Figure 4A – Lung window; streaky air densities (red arrow) are seen anterior to the pericardial space outlining the pulmonary vasculature bilaterally. Figure 4B – Air is seen outlining the descending abdominal aorta and the medial aspect of the spleen with air lucencies (cyan arrow) also seen in the gastro-hepatic space, the anterior pre-peritoneal space, and adjacent to the right diaphragmatic crus. Figure 4C – Axial image at the level of the L2 vertebral body showing air foci (cyan arrow) in the extra-peritoneal space. Figure 4D – At the level of the L5 vertebral body with air foci seen in the extra-peritoneal space. A few air foci are seen anterior to the left psoas, which is the major muscle. Figure 4E – Axial place image through the sacro-iliac joints showing abundant air foci (pink arrow) in the para-rectal space extending superiorly. Figure 4F – At the level of the femoral heads, the air is noted (pink arrow) in the para-rectal space, with few air foci seen in the anterior pre-peritoneal space.

A 39-year-old man with no comorbidities, who had undergone a haemorrhoidectomy, had presented with pain in the perianal area three days after the surgery. On examination, the abdomen appeared soft and non-tender, while a digital rectal examination showed tenderness at the operation site (Figure 4).

Patient 3

**Figure 5:**
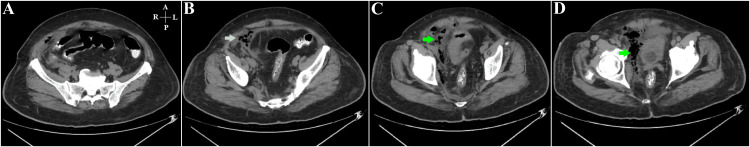
Patient 3 – CT images of the abdomen and pelvis in the axial plane Figure 5A – At the level of the sacro-iliac joints; Air-containing collection was noted in the inferior aspect of the right anterior, lateral abdominal wall, in the pre-peritoneal space. Figure 5B – S2-S3 level; air-containing collection (cyan arrow) in the pre-peritoneal space of the right antero-inferior abdominal wall with fascial thickening and regional fat stranding. Figure 5C – Collection with air foci (green arrow) within is seen in the right anterior and posterior extra-peritoneal spaces. Figure 5D – Axial CT images of the pelvis; an air-containing collection (green arrow) is seen on the right, medial to the obturator internus, lateral to the rectum, extending anteriorly adjacent to the urinary bladder.

A 50-year-old male with type 2 diabetes mellitus and hypertension presented with symptoms of fever, chills, and lower abdominal pain. The right iliac fossa was tender upon abdominal examination, and there was no palpable mass. There was no induration or tenderness during the digital rectal examination (Figure 5).

Patient 4

**Figure 6:**
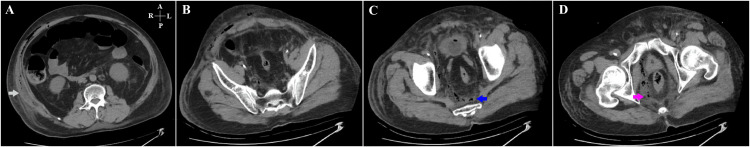
Patient 4 - CT images of the abdomen and pelvis in the axial plane Figure 6A – An air-containing collection (cyan arrow) is seen in the right abdominal wall in the intermuscular plane. Intra-peritoneal and retroperitoneal fat stranding is seen. Figure 6B – Image at the level of the sacro-iliac joints; Air-containing collection seen in the inferior aspect of the anterior abdominal wall with stranding of the overlying fat in the subcutaneous plane. Multiple air foci are seen in the pelvis, posterior to the mesorectal fascia, with regional fat stranding and fascial thickening. Figure 6C – Multiple air foci (blue arrow) are seen in the pelvis in the extra-peritoneal space. Figure 6D –At the level of the pubic symphysis; An air-containing collection (pink arrow) is seen in the pelvis on the right of the midline with fascial thickening and stranding of the para-rectal fat

A 45-year-old male patient with no comorbidities who was diagnosed with perianal abscess underwent incision and drainage according to the treatment plan. A week later, the patient arrived at our hospital in septic shock, experiencing pain in the perianal region along with a fever. On examination, he had tachycardia and was in hypotension. Furthermore, abdominal examination revealed skin changes in the lower abdomen, and on digital rectal examination, purulent discharge was noted from the site of a previous operation (Figure 6).

Patient 5

**Figure 7:**
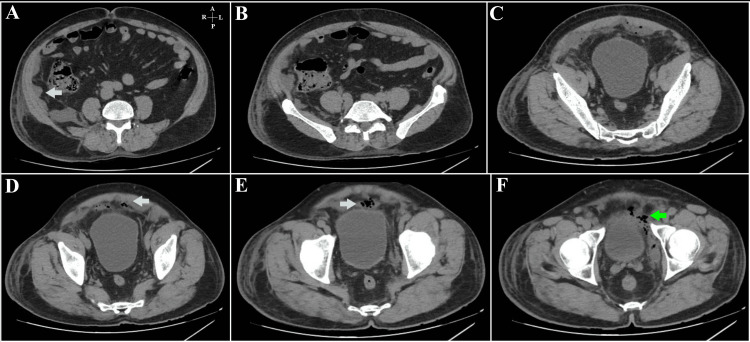
Patient 5- CT images of the abdomen and pelvis in the axial plane Figure 7A– Through the L3 vertebral body; a fluid density collection (cyan arrow) is seen in the extra-peritoneal space on the right with peritoneal thickening and stranding of fat. Figure 7B– Extra-peritoneal fat stranding is noted on the right, with a collection in the extra-peritoneal space posterior to the ascending colon. Figure 7C– An air-containing collection is noted in the pre-vesical and right anterior abdominal wall with peritoneal thickening and extensive regional fat stranding. Figure 7D– Axial image of the pelvis with an air-containing collection (cyan arrow) seen posterior to the rectus abdominis muscle, anterior to the urinary bladder. Figure 7E– A loculated collection with multiple air foci (cyan arrow) is seen anterior to the urinary bladder with fascial thickening and fat stranding. Figure 7F– At the level of the femoral heads; a heterogeneous air-containing collection (green arrow) is noted in the pre-vesical and left hemi-pelvis, medial to the anterior aspect of the obturator internus muscle.

A 55-year-old male patient with no comorbidities came in with discomfort in the right lumbar region that had been persisting for five days. On abdominal examination, tenderness was noted in the right lumbar region (Figure 7).

Patient 6

**Figure 8:**
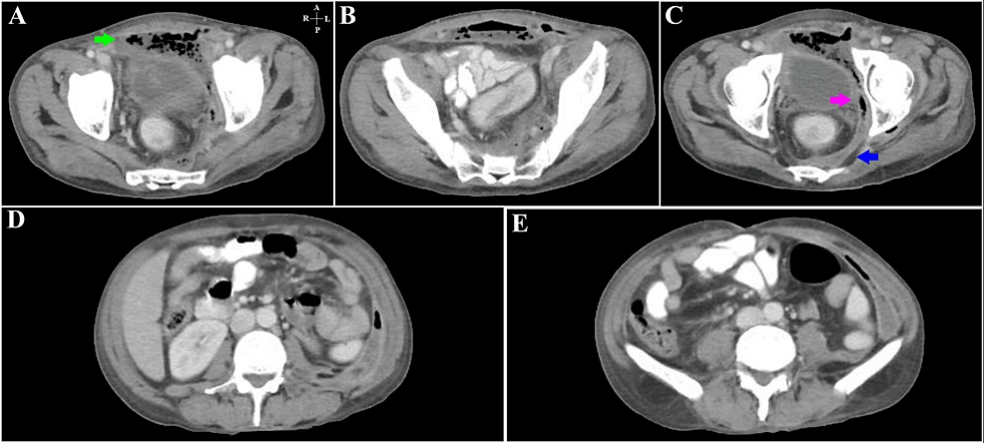
Patient 6 - Contrast-enhanced CT of the abdomen and pelvis in the axial plane Figure 8A – Axial images through the pelvis with a large air-containing collection (green arrow) seen anterior to the urinary bladder and along the left hemi-pelvic wall. A few air foci are also seen in the left rectus abdominis muscle. Figure 8B – A peripherally enhancing, air-containing collection is noted in the pre-peritoneal space posterior to the rectus abdominis muscle and the presacral space. Figure 8C – At the level of the femoral heads; a peripherally enhancing air-containing collection (pink arrow) is seen along the left hemi-pelvis extending anteriorly in the extra-peritoneal space. The collection (blue arrow) is also seen extending posterior to the rectum. Stranding of surrounding fat is noted. Figure 8D – L3-L4 level; An air-containing peripherally enhancing collection is seen along the left lateral abdominal wall with stranding of fat medially. Figure 8E – The peripherally enhancing collection is seen along the left abdominal wall medial to the transversus abdominis muscle with air within.

A 45-year-old patient with type 2 diabetes mellitus who had initially presented with a bilateral ischiorectal abscess underwent incision and drainage. The patient had persistent discharge from the surgical site in the postoperative period and pain in the left lumbar region. There was tenderness and purulent discharge from the operative site on digital rectal examination (Figure 8).

## Discussion

The pelvic extraperitoneal spaces are difficult to access for clinical examination, and hence, the pathologies in these regions remain occult for a long time with prolonged illness. Diagnostic delay and inadequate drainage are common, resulting in prolonged sepsis and associated high morbidity and mortality [1]. Understanding the pelvic extraperitoneal spaces and their communication is very important to identify the source and spread of infection and, hence, with the appropriate patient management. In our study, we have attempted to understand the anatomy of pelvic extraperitoneal spaces by cadaver dissections and correlating it with CT images of patients who presented to us with extraperitoneal abscesses.

The umbilicovesical fascia (Figure 1A-1) extends from the umbilicus to surround the urinary bladder (Figure 1B-2), urachus, and obliterated umbilical arteries and forms the peri vesical space similar to the renal fascia, which envelops the kidney to form the perirenal space. It surrounds the urachus, medial umbilical ligament, and urinary bladder. O'Connell et al., in their study on CT of pelvic extraperitoneal spaces using cadavers, demonstrated communication between perivesical, prevesical, and rectal extraperitoneal spaces [4]. The prevesical space lies between the umbilicovesical fascia and the transversalis fascia, lies anterolateral to the urinary bladder (Figure 2A-1), and is the largest potential compartment in the pelvic extraperitoneal space [5]. The extraperitoneal collection in the pelvis can extend into the retroperitoneal spaces via the prevesical space, and the prevesical space is continuous with pre-sacral space, rectus sheath, and femoral sheath. The prevesical space is continuous with the infrarenal retroperitoneum [6]. This intercommunication can be seen in the CT images of the abovementioned patients. The pre-sacral space is a retroperitoneal space located between the parietal peritoneum of the posterior abdominal wall and the sacrum [7]. Superiorly, the space extends up to the peritoneal reflection and inferiorly to the retro sacral fascia, which passes forward from the S-4 vertebra to the rectum ~3 to 5 cm proximal to the anorectal junction [8]. It contains loose areolar and connective tissue, within it are the superior hypogastric plexus, hypogastric nerves, and portions of the inferior hypogastric plexus [7].

The rectal extraperitoneal space is the space between the wall of the rectum below the rectal peritoneal reflection and parietal pelvic fascia. Around the rectum is a distinct compartment of extraperitoneal tissue called the mesorectum. The mesorectal fascia encloses this mesorectum and surrounds the rectum to form the perirectal space similar to the peri vesical space for the urinary bladder. The mesorectum and its fascia are separated from the pelvic wall by loose areolar (fatty) tissue to form the pararectal space [9].

The extraperitoneal space's continuity with the thorax above and the lower limbs below also provides access for the bidirectional spread of diseases involving these sites [9]. This could explain the pneumomediastinum in one of our patients undergoing an open haemorrhoidectomy.

One of the frequent surgical emergency conditions encountered in the emergency department is anorectal abscesses, which are typically treated with antibiotics, incision, drainage and a rare complication of an anorectal abscess is the upward spread of infection into the supralevator and extraperitoneal spaces. These abscesses tend to develop below the puborectalis muscle, which acts as a barrier limiting the cephalad extension [3]. Supralevator abscesses are the least common, occurring in only 1% to 9% of patients, and their spread across specific anatomic spaces is a very rare complication that could potentially result in sepsis and death [10]. The spread of infection into the perianal region highlights the complexity of the anatomic planes and the connections of the retroperitoneal and extraperitoneal spaces [3]. It is crucial to understand the symptomatology in these individuals; in our study, three patients diagnosed with an ischiorectal abscess who later underwent incision and drainage experienced pain in the abdomen and persistent discharge from the operation site. Among the patients diagnosed with ischiorectal abscesses, pain in the abdomen indicates extraperitoneal inflammation [10]. Given the diffuse nature of the suppuration rather than a distinct collection, extraperitoneal surgical drainage is considered the gold standard treatment [3], which is the treatment provided to the patients in our case series. In abscesses with extensive pre- or retroperitoneal extensions, access to the peritoneal cavity must be avoided due to the high risk of contamination and secondary peritonitis [10]. Konstantinos et al. mention that early recurrences, after incision and drainage, are a major risk factor for abscess expansion, especially if the patient bears comorbidities, such as diabetes [10]. Similarly, in our study, three of the six patients who were diagnosed with extraperitoneal abscess had diabetes mellitus.

Limitations

The interpretation of imaging data and anatomical dissections with the use of cadavers involves a level of subjectivity. Through cadaver dissections, we gained valuable insights into the intricacies of anatomy, although the dissection falls short of demonstrating the interconnections of different spaces within the body.

## Conclusions

The significance of comprehending the communication pathways within these areas, such as the peri-vesical, prevesical, pre-sacral, and pararectal spaces, is underscored by our research findings. These spaces are very vital as they aid in the spread of infection. The loose areolar tissue between these spaces may play a role in transmitting infections, emphasizing the importance of prompt and precise diagnosis.

Our study shows challenges in diagnosing and managing pelvic extraperitoneal abscesses, which may prolong the duration of the disease. Inferring from our findings, we would like to state that patients with anorectal suppurative diseases who remain symptomatic after initial treatment should undergo a CT scan, facilitating quick diagnosis and proper treatment. Surgical extraperitoneal drainage is considered the best treatment modality in patients with an extraperitoneal abscess. The connection between extraperitoneal spaces and other anatomical regions, like the thorax and lower limbs, highlights the possibility of diseases spreading cranially and caudally. Our research also adds to the expanding understanding of pelvic extraperitoneal pathologies and highlights the significance of a collaborative approach in diagnosing and treating them. Improving our knowledge of these anatomical structures can lead to better ways of diagnosing and treating patients with pelvic extraperitoneal complications, ultimately lowering morbidity and mortality related to these conditions.

## References

[1] Crepps JT, Welch JP, Orlando R 3rd (1987). Management and outcome of retroperitoneal abscesses. Ann Surg.

[2] Hamza E, Saeed MF, Salem A, Mazin I (2017). Extraperitoneal abscess originating from an ischorectal abscess. BMJ Case Rep.

[3] Liz A, Madera F, Folonier JC (2022). Extraperitoneal suppuration as a complication of a deep perianal abscess. Case report [Article in Spanish]. Rev Argent Coloproct.

[4] O'Connell AM, Duddy L, Lee C, Lee MJ (2007). CT of pelvic extraperitoneal spaces: An anatomical study in cadavers. Clin Radiol.

[5] Kim SW, Kim HC, Yang DM, Min GE (2013). The prevesical space: Anatomical review and pathological conditions. Clin Radiol.

[6] Korobkin M, Silverman PM, Quint LE, Francis IR (1992). CT of the extraperitoneal space: Normal anatomy and fluid collections. AJR Am J Roentgenol.

[7] Wieslander CK, Rahn DD, McIntire DD, Marinis SI, Wai CY, Schaffer JI, Corton MM (2006). Vascular anatomy of the presacral space in unembalmed female cadavers. Am J Obstet Gynecol.

[8] Hassan I, Wietfeldt ED (2009). Presacral tumors: Diagnosis and management. Clin Colon Rectal Surg.

[9] Mirilas P, Skandalakis JE (2010). Surgical anatomy of the retroperitoneal spaces part II: The architecture of the retroperitoneal space. Am Surg.

[10] Konstantinos PS, Andreas D, Kleoniki K, Dimitrios F (2021). Extraperitoneal spread of anorectal abscess: A case report and literature review. Ann Coloproctol.

